# Long-term follow-up of low-risk branch-duct IPMNs of the pancreas: is main pancreatic duct dilatation the most worrisome feature?

**DOI:** 10.1038/s41424-018-0026-3

**Published:** 2018-06-13

**Authors:** Maria Chiara Petrone, Pietro Magnoni, Ilaria Pergolini, Gabriele Capurso, Mariaemilia Traini, Claudio Doglioni, Alberto Mariani, Stefano Crippa, Paolo Giorgio Arcidiacono

**Affiliations:** 1 Pancreato-Biliary Endoscopy and Endosonography Division, Pancreas Translational & Clinical Research Center, Vita-Salute San Raffaele University, IRCCS San Raffaele Scientific Institute, Milan, Italy; 2 Department of Surgery, Università Politecnica delle Marche, Ancona, Italy; 3 Digestive and Liver Disease Unit, S. Andrea University Hospital, Rome, Italy; 4 Department of Pathology, IRCCS San Raffaele Scientific Institute, Milan, Italy; 5 Pancreas Translational & Clinical Research Center, Division of Pancreatic Surgery, Università Vita-Salute, IRCCS San Raffaele Scientific Institute, Milan, Italy

## Abstract

**Objectives:**

The management of branch-duct IPMN remains controversial due to the relatively low rate of malignant degeneration and the uncertain predictive role of high-risk stigmata (HRS) and worrisome features (WFs) identified by the 2012 International Consensus Guidelines. Our aim was to evaluate the evolution of originally low-risk (Fukuoka-negative) BD-IPMNs during a long follow-up period in order to determine whether the appearance of any clinical or morphological variables may be independently associated with the development of malignancy over time.

**Methods:**

A prospectively collected database of all patients with BD-IPMN referring to our Institute between 2002 and 2016 was retrospectively analyzed. Univariate and multivariate analysis of association between changes during follow-up, including appearance of HRS/WFs, and development of malignancy (high-grade dysplasia/invasive carcinoma) was performed.

**Results:**

A total of 167 patients were selected for analysis, and seven developed malignant disease (4.2%). During a median follow-up time of 55 months, HRS appeared in only three cases but predicted malignancy with 100% specificity. Worrisome features, on the other hand, appeared in 44 patients (26.3%). Appearance of mural nodules and MPD dilatation >5 mm showed a significant association with malignancy in multivariate analysis (*p* = 0.004 and *p* = 0.001, respectively). MPD dilatation in particular proved to be the strongest independent risk factor for development of malignancy (OR = 24.5).

**Conclusions:**

The risk of pancreatic malignancy in this population is low but definite. The presence of major WFs, and especially MPD dilatation, should prompt a tighter follow-up with EUS and a valid cytological analysis whenever feasible.

## Introduction

Pancreatic cystic neoplasms (PCNs) have become very frequent incidental findings especially in the elderly population, thanks to technical improvements and extensive use of cross-sectional imaging^1,2^. Of all PCNs, intraductal papillary mucinous neoplasms (IPMNs) are now the most frequent as their prevalence has been steadily increasing during the last decade^3,4^.

IPMNs are well-acknowledged precursor lesions for pancreatic ductal adenocarcinoma (PDAC), with a markedly different risk of malignant degeneration based on the pattern of ductal involvement. IPMNs arising in the main pancreatic duct (MD-IPMNs) have a significant malignant potential and an indication for surgical resection^5,6^. However, the vast majority of IPMNs arising in side ductal branches (BD-IPMNs) degenerate far less frequently, with a rate of ~3.7% and an estimated annual risk of 0.7% for patients undergoing non-operative management^7^. Although BD-IPMNs do represent a precancerous condition offering the opportunity to cure a pancreatic neoplasm before an incurable invasive cancer develops, their rate of degeneration is so low that the risks associated with pancreatic surgery might outweigh the benefits of resection. Therefore, the current management of BD-IPMNs consists of surveillance with therapeutic options based on the presence of specific morphologic features that are associated with the risk of malignancy.

International Consensus Guidelines (ICG) for the evaluation and management of IPMN were published in 2006 and later revised in 2012 after the 14^th^ meeting of the International Association of Pancreatology in Fukuoka, Japan^5^. On that occasion, predictors of malignancy were stratified into two sets of variables bearing a different risk of degeneration, namely high-risk stigmata (HRS) and worrisome features (WFs). Similar definitions of predictive factors were proposed by the European guidelines for the management of PCNs in 2013^6^. These predictors mostly consist of basic morphological and clinical criteria. HRS include the presence of a mural nodule with demonstrated enhancement on either cross-sectional imaging or endoscopic ultrasound (EUS), jaundice, and a MPD dilatation ≥10 mm, and their presence warrants surgical resection in fit patients. The presence of less pronounced changes, i.e., BD-IPMN size ≥3 cm, mural nodules without demonstrated enhancement, cyst wall thickening, MPD dilatation between 5 and 9 mm and episodes of acute pancreatitis, define the diagnosis of WFS. In such cases the current guidelines suggest an evaluation with EUS, the imaging technique that has the highest resolution of the pancreas, offers the chance of fine-needle aspiration (EUS-FNA) and could help the selection of patients with an indication for surgical resection.

The problem clinicians are facing today is that diagnosis with FNA is reported to be successful in half of cases at most^8^ and decision making mainly relies on imaging alone. Management of BD-IPMN is also controversial because the natural history of the disease and the effective role of each of the HRS/WFs in predicting the risk of malignant degeneration have yet to be fully understood. Considering that most IPMNs are incidental findings, it is not infrequent to diagnose cysts that already harbor at least one of the WFs (especially a size ≥3 cm), thus limiting the possibility to evaluate their role as predictive factors during the natural history of the disease.

The present study aims at evaluating the role of possible predictive variables of malignancy development in a carefully selected population of patients with BD-IPMNs which are “naïve” (i.e., Fukuoka-negative for the presence of HRS or WFs) at the time of diagnosis. The primary endpoint is to evaluate the association between development of malignancy and any clinical and radiological features, with particular regard to morphological changes during follow-up including the appearance of HRS and WFs defined as per the 2012 ICG.

## Methods

### Study design

This is a single-center retrospective cohort study performed on a prospectively collected database. We initially interrogated the database for patients enrolled in the Gastroenterology and Gastrointestinal Endoscopy Unit of San Raffaele Scientific Institute, Milan, Italy, who were at least once classified as having a certain or highly probable diagnosis of branch-duct IPMN between September 2002 and December 2016. Certain diagnosis was defined by a conclusive result of EUS-FNA cytology or histological examination of surgical specimens. A highly probable diagnosis was considered in presence of cystic lesions ≥5 mm communicating with the MPD, as clearly demonstrated by MRI/MRCP and/or EUS^9^.

Only patients with BD-IPMNs without HRS or WFs (i.e., Fukuoka-negative) were selected for the analysis, and a follow-up endpoint of at least 24 months was established for patients who were managed non-operatively. Criteria for exclusion were therefore: (a) the presence of HRS or WFs at the time of diagnosis; (b) an inadequate follow-up, defined as the lack of any follow-up examinations, a follow-up time <24 months, or a lack of continuity in the follow-up (i.e., gaps greater than 24 months between consecutive examinations); (c) misdiagnosis, established either clinically by consecutive examinations or histologically on surgical specimens.

Data collected at the time of diagnosis included sex, age, family history of PDAC, history or presence of symptoms (abdominal pain, nausea and/or vomiting, unintentional weight loss), and baseline morphological features: unifocal/multifocal disease, main cyst location, main cyst size, and MPD caliber. Follow-up was performed by means of EUS, cross-sectional imaging and/or trans-abdominal ultrasound (US) depending on physician’s preference, and the results were recorded in a dedicated database. The use of US was reserved for consistently low-risk BD-IPMNs showing no changes over a considerably long span of time, so that the relatively low accuracy of the technique would not influence relevant outcomes.

The appearance of a defined pancreatic solid neoplasm, HRS or WFs during the follow-up were the main outcomes of interest. The following changes occurring during the follow-up were also recorded: (a) cyst size growth ≥5 mm between consecutive examinations; (b) appearance of new cysts; (c) size growth of any mural nodules ≥2 mm; and (d) appearance of new symptoms. The finding of high-grade atypia on EUS-FNA, when performed, was not equated to other clinical and radiological ICG criteria but rather considered as a definitive diagnosis of malignancy, and its role was assessed separately. In operated patients, lesions were classified as benign (low-/moderate-grade dysplasia) or malignant (high-grade dysplasia or invasive carcinoma) by histological examination.

### Statistical analysis

All statistical analyses were performed using IBM SPSS Statistics for Windows, Version 23.0 (Armonk, NY: IBM Corp). Kaplan–Meier estimates were used to describe progression-free survival in our population, considering appearance of WFs, and development of malignancy as the event of interest, respectively. Subgroup comparisons on categorical variables were performed using Pearson’s χ^2^ test or Fisher’s exact test as appropriate. Features showing a significantly different frequency between subgroups were selected for analysis of association with malignancy. Binary logistic regression was used for univariate and multivariate analysis with a forward selection model. Results were considered to be statistically significant when *p* values were <0.05. Collinearity between significant predictors was tested by calculating the Spearman correlation coefficient and results <0.8 were considered acceptable.

## Results

### Descriptive statistics

**Fig. 1:**
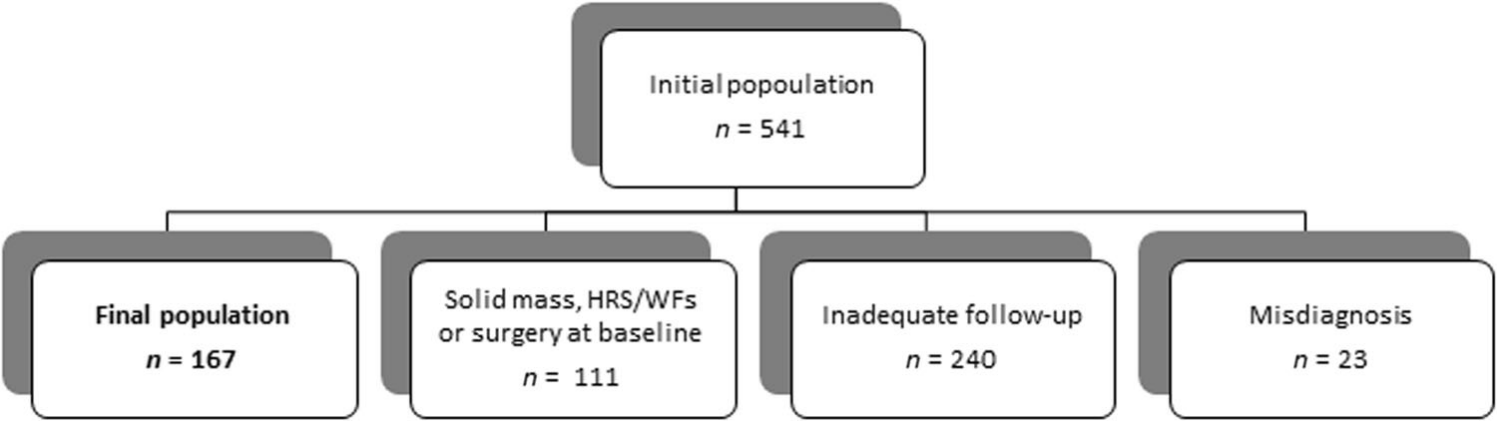
Flow diagram illustrating the exclusion criteria applied to select cases for analysis. HRS high-risk stigmata, WFs worrisome features

From a total of 541 patients resulting from our initial research, 167 patients with BD-IPMNs without HRS or WFs and with available follow-up data were included in the study (see Fig. 1). Patients’ characteristics and baseline features are summarized in Table 1.

**Table 1:** Demographic and clinical variables, diagnostic work-up, and baseline features of our low-risk branch-duct IPMN population

Baseline variables	Value
Age at diagnosis, median with range (years)	66 (33–84)
Sex	
Female, No. (%)	105 (62.9%)
Male, No. (%)	62 (37.1%)
Family history of PDAC, No. (%)	6 (3.6%)
History or presence of symptoms, No. (%)	31 (18.6%)
Pain, No. (%)	25 (15.0%)
Nausea/Vomiting, No. (%)	2 (1.2%)
Weight loss, No. (%)	4 (2.4%)
Incidental finding, No. (%)	136 (81.4%)
Unifocal disease, No. (%)	61 (36.5%)
Multifocal disease, No. (%)	106 (63.5%)
Two cysts, No. (%)	36 (21.6%)
≥three cysts, No. (%)	70 (41.9%)
Main BD cyst location	
Head and uncinate process, No. (%)	72 (43.1%)
Neck and body, No. (%)	78 (46.7%)
Tail, No. (%)	17 (10.2%)
Main BD cyst size, mean ± SD (mm)	14.5 ± 5.7
MPD caliber, mean ± SD (mm)	2.6 ± 0.8

*BD* branch-duct, *EUS* endoscopic ultrasound, *FNAC* fine-needle aspiration cytology, *PDAC* pancreatic ductal adenocarcinoma, *SD* standard deviation.

Only 31 patients presented with symptoms, which makes most of the diagnosed BD-IPMNs “incidentalomas” (81.4%). Single lesions were found in 61 patients, whereas 106 patients (63.5%) had multifocal disease. The mean lesion size was 14.5 ± 5.7 mm, ranging from 5 to 29 mm (i.e., the cut-offs for diagnosis of BD-IPMN and the consideration of cyst size as “worrisome”, respectively). The mean MPD diameter was 2.6 ± 0.8 mm. These values comply with our selection criteria for low-risk BD-IPMNs.

The median follow-up time was 55 months ranging up to a maximum of 134 months (Table 2). Overall changes were reported in 97 patients (58.1%) after a median time from diagnosis of 25 months. Such changes mostly consisted of dimensional growth (35.3%) or appearance of new lesions (33.5%). HRS appeared in only three patients (1.8%) after a median follow-up time of 45 months. WFs, on the other hand, appeared in 44 patients (26.3%) after a median time of 26 months, with an extremely wide range (4–132 months). EUS-FNA was performed in 63 out of 167 patients (37.7%), and a positive cytology result (high-grade atypia) was obtained in four cases (2.4%).

**Table 2:** Results of follow-up including frequency of changes and appearance of high-risk stigmata/worrisome features

Follow-up Variables	Value
Follow-up time, median with range (months)	55 (13–134)
Changes during follow-up, No. (%)	97 (58.1%)
Appearance of high-risk stigmata, No. (%)	3 (1.8%)
Enhanced mural nodule, No. (%)	1 (0.6%)
MPD caliber ≥10 mm, No. (%)	1 (0.6%)
Jaundice, No. (%)	1 (0.6%)
Time to develop HRS, median with range (months)	56 (45–60)
Appearance of worrisome features, No. (%)	44 (26.3%)
Cyst size ≥3 cm, No. (%)	21 (12.6%)
Cyst wall thickening, No. (%)	12 (7.2%)
Mural nodule, No. (%)	13 (7.8%)
MPD caliber 5–9 mm, No. (%)	10 (6.0%)
Acute pancreatitis, No. (%)	4 (2.4%)
Time to develop WFs, median with range (months)	26 (4–132)
Additional features	
Cyst growth ≥5 mm, No. (%)	59 (35.3%)
Appearance of new cysts, No. (%)	56 (33.5%)
Mural nodule growth ≥2 mm, No. (%)	2 (1.2%)
Appearance of new symptoms, No. (%)	7 (4.2%)
Diagnosis of high-grade atypia on EUS-FNA, No. (%)	4 (2.4%)

*EUS-FNA* endoscopic ultrasound-guided fine-needle aspiration, *HRS* high-risk stigmata, *MPD* main pancreatic duct, *WF* worrisome feature

**Fig. 2:**
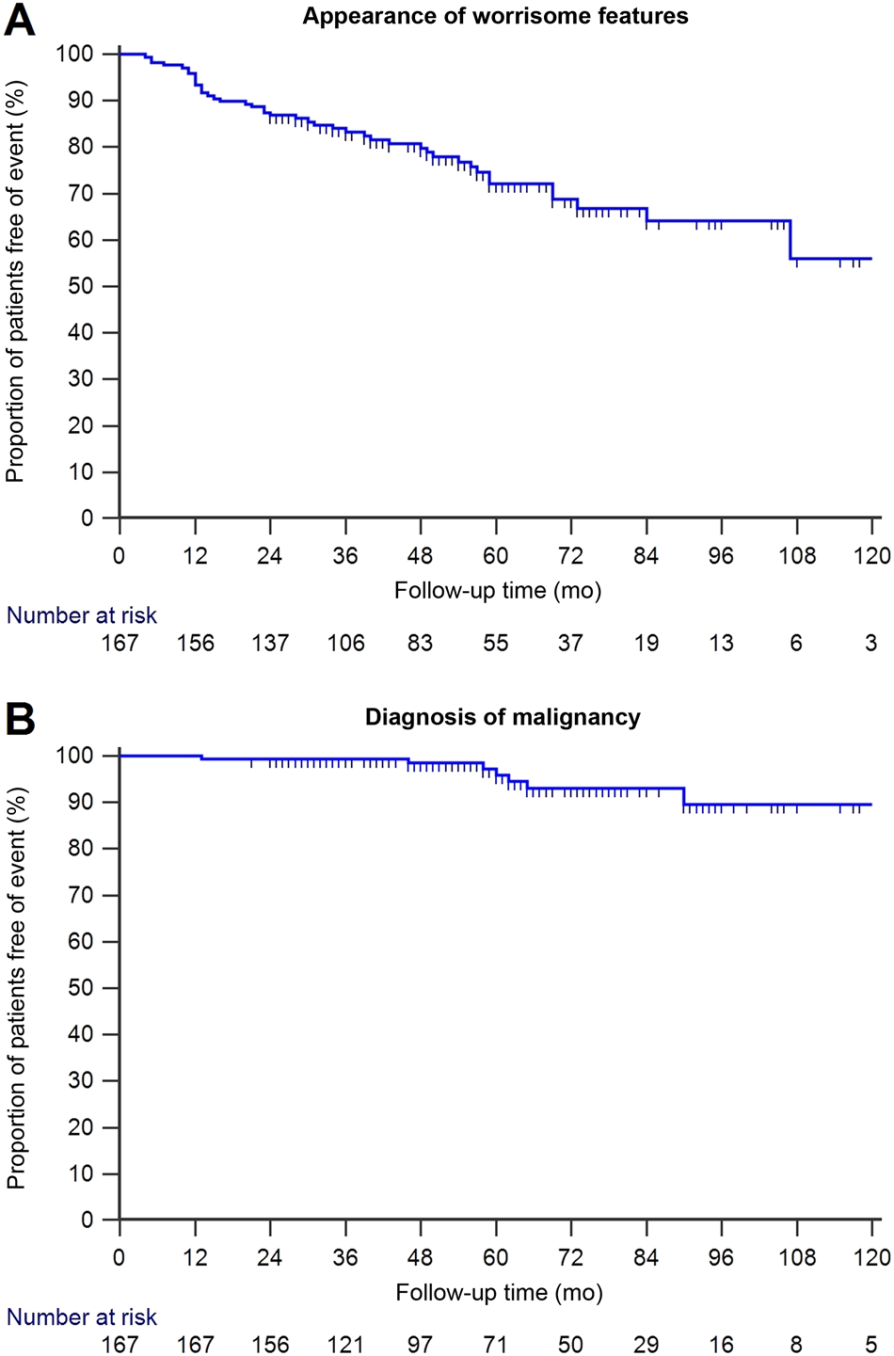
Kaplan–Meier curves showing progression-free survival in our low-risk branch-duct IPMN population over a 10-year period. The events of interest were appearance of worrisome features (**a**) and development of malignancy (**b**), respectively

Surgery was performed in eight patients (4.8%) after a median follow-up time of 52 months (range 13–90 months). The histological examination demonstrated benign disease in two of them, whereas malignancy was diagnosed in six cases (3 high-grade dysplasia, 3 carcinoma), which corresponds to 75% of operated patients. A single patient developed an inoperable solid mass, bringing the count to seven malignancies (4.2% of the overall study population). All other patients without a histological demonstration of malignant disease served as the control group for the analysis of association with malignancy. The progression-free survival curves for the appearance of WFs and the diagnosis of malignancy during follow-up are shown in Fig. 2.

In seven out of eight cases, EUS had a primary role in establishing the main indications for surgery: presence of mural nodules (5) with or without finding of high-grade atypia on EUS-FNA (3 out of 5), presence of a solid mass associated with jaundice and MPD dilatation (1), and rapid MPD caliber enlargement to ≥10 mm documented over two consecutive EUS examinations (1). The remaining case consisted of a large (40 mm) and rapidly growing (>5 mm/year) cyst studied by MRI/MRCP. Although most operated patients had BD-IPMNs with mural nodules detected by EUS, two cases of false positivity for malignancy and overtreatment were also registered in this subgroup (1 low-grade dysplasia, 1 intermediate-grade dysplasia). In those two cases, EUS-FNA had not been performed. EUS was also able to detect solid masses that had been described by contrast-enhanced CT as undefined parenchymal changes in two cases, consisting of one case of operated IPMN-carcinoma and one case of inoperable PDAC distinct from IPMN which developed over a 6-month time span. Further details about preoperative and operative data are listed in Table 3.

**Table 3:** Preoperative and operative details regarding nine patients who underwent surgery and/or received a final diagnosis of malignancy

Pt. #	Age (y)	Year of diagnosis	Time from diagnosis (mo)	Indication	Primary role of EUS	FNAC	Procedure	Histology
1	63	2005	13	WFs: nodule, cyst size ≥3 cm, cyst wall thickening	Yes	HGA	TP	IPMN-PDAC
2	65	2005	90	WFs: nodule, cyst wall thickening	Yes	HGA	DP	HGD (gastric type)
3	66	2007	58	HRS: jaundice WF: MPD 5–9 mm	Yes (detected solid mass)	LGA	PD	IPMN-PDAC
4^a^	72	2009	21	WFs: nodule, acute pancreatitis	Yes	—	DP	LGD (gastric type)
5^a^	46	2010	21	WFs: nodule, cyst size ≥3 cm	Yes	—	PD	IGD (intestinal type)
6^b^	81	2010	50	WF: MPD 5–9 mm	Yes (detected solid mass)	HGA	—	—
7	52	2011	60	WF: cyst size ≥3 cm (40 mm) Cyst size growth >5 mm/y	No	—	PD	HGD (intestinal type)
8	60	2011	46	WF: MPD 5–9 mm rapidly escalated to HRS: MPD ≥10 mm	Yes	—	PD	IPMN-PDAC
9	57	2011	62	HRS: enhancing nodule	Yes	HGA	PD	HGD (gastric type)

The indications for surgery were established by endoscopic ultrasound in seven out of eight cases. Presence of any other worrisome features was reported, although they would not drive surgical decision per se

*DP* distal pancreatectomy, *EUS* endoscopic ultrasound, *FNAC* fine-needle aspiration cytology, *LGA* low-grade atypia, *LGD* low-grade dysplasia, *HGA* high-grade atypia, *HGD* high-grade dysplasia, *HRS* high-risk stigmata, *IGD* intermediate-grade dysplasia, *MPD* main pancreatic duct, *PD* pancreaticoduodenectomy, *PDAC* pancreatic ductal adenocarcinoma, *TP* total pancreatectomy, *WF* worrisome feature

^a^Surgical overtreatment of patients with low- to intermediate-grade dysplasia on histological examination

^b^Inoperable patient with pancreatic ductal adenocarcinoma distinct from IPMN

### Analysis of association with malignancy

Among all features described during the follow-up, a statistically significant difference in frequency in patients with malignant disease was found for presence of MPD dilatation (*p* = 0.005 for MPD 5–9 mm, *p* = 0.042 for MPD ≥10 mm), mural nodules (*p* = 0.011 for mural nodules without demonstrated enhancement, *p* = 0.042 for enhanced mural nodules, *p* = 0.002 for mural nodule growth) and jaundice (*p* = 0.042). A cyst size ≥3 cm in particular did not impact the frequency of malignancy (*p* = 0.215) (Table 4).

**Table 4:** Different frequency of changes and appearance of high-risk stigmata/worrisome features in patients with malignant disease compared to controls

Variable	Control Group (*n* = 160)	Malignancy (*n* = 7)	*p* value
High-risk stigmata	0	3	
Enhanced mural nodule	0	1	**0.042**
MPD caliber ≥10 mm	0	1	**0.042**
Jaundice	0	1	**0.042**
Worrisome features	37	7	
Cyst size ≥3 cm	19	2	0.215
Cyst wall thickening	10	2	0.081
Mural nodule	10	3	**0.011**
MPD caliber 5–9 mm	7	3	**0.005**
Acute pancreatitis	4	0	1.000
Additional features			
Family history of PDAC	6	0	1.000
History of symptoms	31	0	0.351
Multifocal disease	101	5	1.000
Cyst growth	57	2	1.000
Appearance of new cysts	56	0	0.097
Mural nodule growth	0	2	**0.002**
New symptoms	6	1	0.263
High-grade atypia on EUS-FNA	0	4	<**0.001**

*p* values refer to subgroup comparisons with Pearson’s χ^2^ test or Fisher’s exact test

*EUS-FNA* endoscopic ultrasound-guided fine-needle aspiration, *PDAC* pancreatic ductal adenocarcinoma

*p* values which were <0.05 implying statistical significance were highlighted in bold.

Significant variables were included in the univariate analysis adjusted for sex and age. The role of HRS was clear as they predicted malignancy with 100% specificity, but the fact that each of them was present in only one patient spoiled their statistical power in logistic regression. Therefore, only mural nodules and MPD dilatation 5–9 mm were selected by the forward logistic model in multivariate analysis. As discussed above, the role of EUS-FNAC was assessed separately. All four cases of cytological high-grade atypia were proven to harbor malignant disease, which made cytology (when available) the strongest predictor of malignancy with 100% specificity. Nonetheless, inclusion of a positive cytology result in the multivariate analysis did not alter the model and both mural nodules and MPD dilatation remained as independent risk factors associated with the development of malignancy (OR 17.0, *p* = 0.004 and OR 24.5, *p* = 0.001, respectively) (Table 5). A Spearman correlation coefficient <0.8 excluded collinearity between the two predictors. MPD dilatation 5–9 mm resulted to be the strongest predictor for diagnosis of malignancy.

**Table 5:** Logistic regression analysis of possible predictors of malignancy

Variable	Multivariate analysis		
	OR	95% CI	*p* value
Mural nodule	17.02	2.44–118.59	**0.004**
MPD caliber 5–9 mm	24.48	3.40–176.31	**0.001**

Only mural nodules and MPD dilatation 5–9 mm were retained by the forward selection model for multivariate analysis

*CI* confidence interval, *MPD* main pancreatic duct, *OR* odds ratio

*p* values which were <0.05 implying statistical significance were highlighted in bold

## Discussion

The aim of the present study was to retrospectively evaluate the long-term follow-up of patients with originally low-risk (Fukuoka-negative) BD-IPMNs, in order to determine the significance of the appearance of several clinical and morphological variables as predictors of the development of pancreatic malignancy.

Our series confirms the importance of BD-IPMN as a relatively common incidental finding (81.4% of patients were asymptomatic). More than half of our population showed changes over a median follow-up period of ~4.5 years. Most of these changes were proven harmless, consisting of dimensional growth or appearance of new lesions, which were not found to be significant risk factors for malignancy per se and were likely influenced by inter-observer variability and by the resolution of different imaging modalities. Nonetheless, 4.2% of patients developed a malignant disease during follow-up, in some cases even after a long time span. Therefore, as recently reported elsewhere^10,11^, there is currently insufficient data to safely cease surveillance of BD-IPMN, no matter how stable or innocuous the lesion may appear. At the same time our study shows that many BD-IPMNs do not progress over time, so intensive follow-up protocols may prove not to be cost-effective.

Though not specifically aimed at comparison of guidelines, the present study paves the way for validation of the 2012 International Consensus Guidelines. Our analysis of HRS as predictors of malignancy provided significant results but was hampered by the little proportion of patients showing such features (3 out of 167). This may be informative by itself, as it shows that very few patients with apparently innocuous BD-IPMNs are diagnosed with HRS over time.

Cyst size ≥3 cm is generally considered the most controversial worrisome feature^12^. There is no shortage of studies denying a role for this variable as a risk factor for malignant degeneration in BD-IPMNs without other WFs^13,14^. However, recent meta-analyses present cyst size ≥3 cm as a significant risk factor, with values of OR widely ranging from 2.3^15^ to 62.4^16^. In our cohort, trespassing the 3-cm threshold was found to have no significant association with malignancy. This would support the consideration of cyst size as the weakest morphological predictor of malignancy when using the present 3-cm cut-off set by the ICG. Nonetheless, there was a single case of BD-IPMN displaying only a large cyst size as WF that proved to harbor malignancy. The cyst had reached a maximum diameter of 40 mm and had shown a >5 mm/year growth rate prior to surgery. This fact remains noteworthy and prompts the choice of a different cut-off and/or inclusion of growth rate as a collateral parameter as advocated by the European guidelines^6^, as well as by the latest revision of the ICG in 2017^17^.

The appearance of mural nodules and the increase in MPD diameter during the follow-up were the only WFs, which were selected by our forward logistic model and were found to be significantly associated with development of malignancy both in univariate and multivariate analysis (*p* = 0.001 and *p* = 0.004, respectively). Results reported by Jang et al. in a large surgical series (*n* = 350) were very similar to ours and identified a significant association for MPD dilatation >5 mm, presence of a mural nodule and cyst wall thickening in univariate analysis (all *p* values <0.001). MPD dilatation and mural nodules were confirmed by multivariate analysis (HR 4.54, *p* < 0.001 and HR 6.26, *p* < 0.001, respectively)^18^. A recent meta-analysis by Ricci et al. found the highest values of pooled diagnostic odds ratio (DOR) for jaundice (6.3), presence of mural nodules (4.8), cyst wall thickening (4.2), and MPD dilatation (4.0)^19^. Similarly, another meta-analysis by Kim et al. found the highest pooled DOR for mural nodules (6.0) followed by MPD dilatation (3.4), and thickened/enhancing cyst walls (2.3). Therefore, the authors advocated a more aggressive approach to mural nodules and a watchful waiting for the other features^15^. When compared to these meta-analyses, our results indicate that MPD dilatation is the strongest predictor of malignancy with an odds ratio which is markedly higher than those of other examined factors (OR 24.5).

The finding of mural nodules on EUS represented the most common indication for surgery in our cohort. The unparalleled sensitivity of EUS in detecting nodules is now widely acknowledged and was recently underlined by Ridtitid et al. in a large single-center study of 364 patients with BD-IPMN, where mural nodules identified by EUS were missed by CT/MRI in 28% of cases in the malignant group^20^. However, a pitfall lies in the classification of nodules as enhanced (HRS) or non-enhanced (WF) with EUS, as the use of intravenous ultrasound contrast agents was introduced relatively recently and is far less routinary than it is for cross-sectional imaging. Considering that our cohort encompasses cases studied more than a decade ago, it is likely that at least some of the nodules (presumably, the ones in the subgroup of operated patients) would have been found positive had an enhancement study been performed. The importance of demonstrating nodule enhancement in an attempt to increase the positive predictive value (PPV) of this predictor is emphasized by the slight change made in the 2017 revision of the ICG. Only enhanced mural nodules on cross-sectional imaging are now mentioned, and their classification as HRS or WF is now based on nodule size with a 5-mm cut-off^17^.

The role of MPD dilatation as a predictor of malignancy directly relates to the controversy that lies in the definition of this variable both as a worrisome feature for BD-IPMN and a criterion to define mixed-type IPMN according to the 2012 ICG. If we consider that mixed IPMN shares the high rates of malignant degeneration of MD-IPMN and should be similarly committed to surgical resection, the appearance of MPD dilatation in a lesion formerly diagnosed as BD-IPMN leads to a therapeutic dilemma. A dilatation greater than 10 mm bears a markedly higher risk of malignancy, but it is a late sign which is actually found in very few cases. On the other hand, a dilatation above 5 mm is a far more frequent finding, but its meaning is not univocal and its PPV for malignancy is limited^21^. This inverse relationship between feature prevalence and PPV was recently confirmed in a study by Ma et al. considering 239 patients with PCNs (including 163 IPMNs) who underwent surgical resection. In their series MPD dilatation ≥10 mm had a high PPV (72.7%) but was present in only 11 patients, whereas MPD dilatation 5–9 mm had a markedly lower PPV (44.1%) but was a much more frequent finding (59 out of 239 operated patients, 24.7%)^22^. Remarkably, MPD dilatation is currently valued as a risk feature regardless of the underlying cause, which may be direct tumor involvement or ductal hypertension caused by mucin, protein plugs, or focal pancreatitis. The occurrence of pure BD-IPMNs with MPD dilatation but without MPD disease can be demonstrated on pathology only, but this information cannot be obtained by imaging for all patients undergoing surveillance, which represents the most common clinical setting. Crippa et al. recently showed that classifying a BD-IPMN with MPD >5 mm as a mixed-type may lead to significant overdiagnosis of mixed-IPMN (8/93, 8.6%) and overtreatment of otherwise harmless BD-IPMNs (2/93, 2.1%)^23^. On the other hand, minimal MPD involvement may be demonstrated as an incidental finding on histology in many cases of radiologically diagnosed BD-IPMNs, although it may not imply a more aggressive biology than pure BD-IPMNs^24^. If we consider the findings in our cohort, in one case of malignancy an initial MPD dilatation was rapidly followed by progressive enlargement to more than 10 mm. This would strongly suggest active MPD involvement, which was found on histological examination. In the other two cases, a thorough EUS evaluation proved MPD dilatation to result from passive retrograde dilatation caused by a solid mass that had not been clearly defined by previous imaging. These findings would lead to question the consideration of MPD dilatation as an “independent” risk factor rather than a sentinel for the presence of other factors which are eluding present understanding of the clinical condition. The topic remains controversial and warrants further clarification of the role of MPD dilatation in the natural history of the disease.

Differently than clinical and radiological features, EUS-FNA allows to directly assess the presence of malignancy within the specimen, the main limitation of this procedure being its low sensitivity. However, once a representative sample is obtained, its results may be considered as a definitive diagnosis with almost absolute specificity. Indeed, high-grade atypia on cytology was found to be a strong predictor of malignancy with 100% concordance. In the above-mentioned meta-analysis by Ricci et al. a positive cytology result had a DOR of 5.5 and the sensitivity, specificity, PPV, and negative predictive value of EUS-FNAC were estimated to be 34.7%, 87.8%, 56.1%, and 75%, respectively^19^. This would lead to the consideration that the finding of WFs in BD-IPMN patients should prompt all attempts to obtain a valid cytological analysis in order to individualize management.

The present study was not designed for comparison or validation of guidelines. Nonetheless, it may be interesting to consider our results in light of the recently published American Gastroenterological Association Institute guidelines on the diagnosis and management of asymptomatic pancreatic cystic neoplasms^25^. After extensive technical review of the literature^26^, these guidelines proposed a conservative approach with indication for surgery only in the presence of two at least out of three high-risk features (cyst size ≥3 cm, MPD dilatation, solid component) confirmed by EUS and/or in presence of a positive cytology result. Mural nodules and MPD dilatation, which had high pooled specificity values reported in the review (80% and 91%, respectively), are also the two significant risk factors outlined by our study. The authors of the AGA guidelines estimated a specificity of 95% for malignancy using their combination criterion. However, Singhi et al. retrospectively applied them to a study cohort of 41 patients with pancreatic cystic lesions referred for EUS-FNA and available pathology results (including 23 IPMNs), and found only a sensitivity of 62% and a specificity of 79% for malignant disease. In their series, application of the AGA guidelines would have deferred surgery for five out of 11 (45.4%) cases of malignant IPMNs (4 carcinoma, 1 high-grade dysplasia) and would not have prevented unnecessary surgery for two out of 12 cases of IPMN with low-grade dysplasia (16.7%)^27^. Similar results were provided by Ge et al. in a multicenter study of 300 patients with PCNs (including 198 IPMNs), where retrospective application of the AGA guidelines had 83.3% sensitivity and 69.1% specificity for malignancy. Although the guidelines accurately recommended surveillance in 95% of patients, nine invasive cancers (5%) would have been missed^28^. If retrospectively applied to our subgroup of operated patients, the AGA guidelines would not have spared the two patients with low-/intermediate-grade dysplasia from overtreatment because of the copresence of mural nodule and cyst size ≥3 cm in one case and symptoms (acute pancreatitis) in the other. Conversely, two cases of malignancy would have been missed (one case with rapidly growing cyst size and one case with rapidly enlarging MPD caliber). These results would indicate that even just one of the morphological high-risk features may be worrisome per se and the limitation of having at least two to consider surgery may be too strict.

The most important caveat with the AGA guidelines emerges from the advocated surveillance protocol, which is very loose for cysts not classified as being “high-risk”. The proposed follow-up consists of MRI in one year and every two years thereafter for a total of only five years, provided that the cyst remains unchanged. However, recent evidence suggests that HRS and WFs may appear well after this time span. A study on long-term follow-up of BD-IPMN by Pergolini et al. found that 20 out of 363 patients (5.5%) developed malignancies after five years of surveillance. More importantly, malignancies developed in 12 out of 282 patients (4.3%) who had absence of HRS/WFs at the five-year threshold and would develop them later, with a median time of 93 months^29^. Similar results were reported in a multicenter study of IPMNs followed-up for more than five years by Crippa and colleagues^11^. In our cohort, most changes developed within the first five years (the median time was 25 months), but there were also cases of WFs appearing after this time span in previously unchanged BD-IPMNs. Discontinuation of follow-up after five years would have missed eight patients developing WFs, including one case with MPD dilatation and one with appearance of a mural nodule. Although all eight patients were still in follow-up and in the control group for analysis at the time of the study, the authors would recommend against generalizing results and setting a threshold for discontinuation of follow-up.

The present study has major flaws due to its retrospective nature. Our study period encompassed 14 years during which clinical decision making and patient management changed progressively according to publication of guidelines and further understanding of the natural history of the disease. Also, some considerations should be made for a careful interpretation of our results. As expected, the rate of malignancy arising in this “naïve”, low-risk BD-IPMN population was low, providing us with few cases developing the outcomes of interest, and ultimately limiting the power of our statistical analysis. Follow-up was performed by means of EUS in our Unit, but any credible reports of cross-sectional imaging and trans-abdominal ultrasound were accepted as well. It may be argued that US has lower accuracy and cannot be considered equally reliable. However, follow-up with US was only employed for consistently low-risk and unchanged cysts. This approach is somewhat justified by the 2014 AIGO-AISP Italian Consensus Guidelines, which suggest the use of US for single lesions that are clearly visible in alternation with MRI/MRCP^30^. It was not our goal to compare the sensitivity and specificity of different techniques, and we valued actual clinical practice over inter-observer and inter-technique variability.

In conclusion, this study shows that more than half of patients with low-risk BD-IPMN develop changes over a long period of time. Although many of these changes are actually harmless, consisting of cyst size growth and finding of new lesions, over one quarter of patients develop HRS/WFs, and the risk of malignancy in this population remains low but not negligible (4.2%). This would discourage discontinuation of surveillance after five years as proposed by the 2015 AGA guidelines. The appearance during follow-up of WFs as indicated by the 2012 ICG resulted to be worrisome indeed, and would justify a tighter follow-up with EUS. MPD dilatation in particular proved to be the strongest independent factor associated with malignancy. In presence of such features, considering the safety and feasibility of EUS-guided FNA in tertiary referral centers, all efforts should be made to obtain an adequate cytological analysis whenever feasible.

## Study Highlights

### What is current knowledge

- Management of branch-duct IPMN has become more and more conservative.
- Surgical decision mainly relies on the presence of clinical and morphological predictors of malignancy described by different guidelines.
- The 2012 International Consensus Guidelines first stratified such predictors into high-risk stigmata and worrisome features, but their role in defining the risk of pancreatic malignancy remains unclear.

### What is new here

- More than one quarter of patients with initially low-risk BD-IPMN developed high-risk stigmata or worrisome features over time and even after a long follow-up period.
- High-risk stigmata and positive cytology results were rare but associated with malignancy with 100% concordance.
- Among worrisome features, mural nodules and especially MPD dilatation >5 mm proved to be the strongest predictors of pancreatic malignancy.
